# Hidden danger: Unexpected scenario in the vector-parasite dynamics of leishmaniases in the Brazil side of triple border (Argentina, Brazil and Paraguay)

**DOI:** 10.1371/journal.pntd.0006336

**Published:** 2018-04-06

**Authors:** Vanete Thomaz-Soccol, André Luiz Gonçalves, Claudio Adriano Piechnik, Rafael Antunes Baggio, Walter Antônio Boeger, Themis Leão Buchman, Mario Sergio Michaliszyn, Demilson Rodrigues dos Santos, Adão Celestino, José Aquino, André de Souza Leandro, Otacílio Lopes de Souza da Paz, Marcelo Limont, Alceu Bisetto, Jeffrey Jon Shaw, Zaida Estela Yadon, Oscar Daniel Salomon

**Affiliations:** 1 Laboratório de Biologia Molecular, Departamento de Engenharia de Bioprocessos e Biotecnologia, Universidade Federal do Paraná, Curitiba, Paraná, Brazil; 2 Laboratório de Ecologia Molecular e Parasitologia Evolutiva, Departamento de Zoologia, Universidade Federal do Paraná, Curitiba, Paraná, Brazil; 3 SESA- Secretaria de Saúde do Estado do Paraná, Curitiba, Paraná, Brazil; Núcleo de vigilância entomológica de Foz do Iguaçu, 9a regional de saúde, Foz do Iguaçu, Brazil e Núcleo de vigilância entomológica de Maringá - 15a regional de saúde–Maringá, Brazil; 4 Pós-Graduação em Gestão Ambiental, Universidade Positivo, Curitiba, Paraná, Brazil; 5 Departamento de Geociências, Pós Graduação em Saúde e Ambiente, Universidade Federal do Maranhão, São Luiz, Maranhão, Brazil; 6 Prefeitura Municipal de Foz do Iguaçu, Centro de Controle de Zoonoses- CCZ, Foz do Iguaçu, Paraná, Brazil; 7 Laboratório de Análise de Padrões Espaciais e Cartografia Temática (LAPE-CT), Laboratório Pedagógico de Geografia (LABOGEO), Universidade Federal do Paraná, Curitiba, Paraná, Brasil; 8 Departamento de Parasitologia, Instituto de Ciências Biomédicas, Universidade de São Paulo, São Paulo, SP, Brazil; 9 Department of Communicable Diseases and Environmental Determinants of Health, Pan American Health Organization, Washington, D.C. United States of America; 10 INMET–Institute National of Tropical Medicine, Puerto Iguazú, Misiones, Argentina; National Institutes of Health, UNITED STATES

## Abstract

Every year about 3 million tourists from around the world visit Brazil, Argentina and Paraguay´s triple border region where the Iguaçu Falls are located. Unfortunately, in recent years an increasing number of autochthonous canine and human visceral leishmaniasis (VL) cases have been reported. The parasite is *Leishmania* (*Leishmania*) *infantum* and it is transmitted by sand flies (Phlebotominae). To assess the risk factors favorable for the establishment and spread of potential vectors the Centers for Disease Control and Prevention light trap (CDC-light trap) collections were made in the Foz do Iguaçu (FI) and Santa Terezinha de Itaipu (STI) townships and along two transects between them. Our study determined the Phlebotominae fauna, the factors that affect the presence and abundance of *Lutzomyia longipalpis* and *Nyssomyia whitmani*, the presence of *L. infantum* in different sand fly species and which *Leishmania* species are present in this region. *Lutzomyia longipalpis* was the prevalent species and its distribution was related to the abundance of dogs. *Leishmania* *infantum* was found in *Lu*. *longipalpis*, *Ny*. *whitmani*, *Ny*. *neivai* and a *Lutzomyia* sp. All the results are discussed within the Stockholm Paradigm and focus on their importance in the elaboration of public health policies in international border areas. This region has all the properties of stable VL endemic foci that can serve as a source of the disease for neighboring municipalities, states and countries. Most of the urban areas of tropical America are propitious for *Lu*. *longipalpis* establishment and have large dog populations. Pan American Health Organization´s initiative in supporting the public health policies in the border areas of this study is crucial and laudable. However, if stakeholders do not act quickly in controlling VL in this region, the scenario will inevitable become worse. Moreover, *L*. *(Viannia) braziliensis* found in this study supports the need to develop public health policies to avoid the spread of cutaneous leishmaniasis. The consequences of socioeconomic attributes, boundaries and frontiers on the spread of diseases cannot be neglected. For an efficient control, it is essential that urban planning is articulated with the neighboring cities.

## Author summary

Every year about 3 million tourists from around the world visit Brazil, Argentina and Paraguay´s triple border region where the Iguaçu Falls are located. Unfortunately, in recent years an increasing number of autochthonous canine and human visceral leishmaniasis (VL) cases have been reported in this area. Our study determined the Phlebotominae fauna of the region, the factors that affect the presence and abundance of *Lutzomyia longipalpis* and *Nyssomyia whitmani*; the presence of *Leishmania (Leishmania) infantum* in different sand fly species and what *Leishmania* species are present. *Lutzomyia longipalpis* was the prevalent species and its distribution was related to the abundance of dogs. *Leishmania (L*.*) infantum* was found in *Lu*. *longipalpis*, *Ny*. *whitmani*, *Ny*. *neivai* and *Lutzomyia* sp. All the results are discussed within the Stockholm Paradigm and focus on their importance in the elaboration of public health policies in international border areas.

## Introduction

Brazil is ranked among the countries with the highest number of Visceral Leishmaniasis (VL) cases in the world, with 92% of the South American cases (i.e., 3,289 cases in 2015), and 43% of the people at risk of VL [1,2]. Increasing numbers of cases in the country are resulting from the adaptation of rural transmission to urban, a condition that has occurred in many Brazilian regions, especially in the Southeast and Midwest regions of the country [2,3]. Cities such as Belo Horizonte (Minas Gerais state, MG), Araguaína (Tocantins state, TO), Campo Grande (State of Mato Grosso do Sul state), Bauru (Sao Paulo state, SP), Palmas (TO), represent 15% of the VL cases [4]. Consequently, there was an unprecedented acceleration in speed of VL dispersion during the 1990’s and the beginning of the 21^st^ century.

In the southern region of Brazil, the disease has been notified in Rio Grande do Sul since the 2000’s [5]. Although signed in the neighbor countries in the beginning of this century [6–9], the VL was not recorded until recently in the extreme-western of Paraná State, region corresponding to the triple border with Argentina and Paraguay. On the Brazilian side, the first record of *Lu*. *longipalpis* was in Foz do Iguaçu city (FI) in 2012. Subsequently, canine visceral leishmaniasis (cVL) and human (HVL) cases were firstly reported in 2013 and 2016 respectively, and the number of VL cases has been increased in the following years [10–12]. For instance, Thomaz-Soccol et al. [13] showed that about 23.8% dogs in FI presents cVL. In this way, delineating the sand fly fauna´s distribution is essential to understand adequately VL´s status in the region, and to reduce the risk of future epizootics. If *Lu*. *longipalpis* is widely abundant in this region, there is a potential risk that the parasite cycle is established. Moreover, it is critical to assess which environmental conditions provide autochthonous transmission of VL and/or CL, once urban areas of FI and its neighboring cities are expanding in zones with large forest reserves.

The study´s objectives are to assess the above described risks by addressing the following questions: 1) based on the distribution of phlebotomine sand fly species in the urban, peri-urban (or ruro-urban) and rural environment in the western region of Paraná, what are the environmental conditions that allow the installation of *Lu*. *longipalpis* populations? 2) are different *Leishmania* species present sympatrically in this region? 3) are other sand flies hosting *L*. *infantum* in areas with recent reports of VL? 4) what is the prevalence of *Leishmania* spp. in these vectors? This information is important to help understand the risk factors and scenarios that are propitious for the founding of enzootic foci. This work is in part belonging to the IDRC #107577 research project (idrc.ca/en/project/addressing-emergence-and-spread-leishmaniasis-borders-argentina-brazil-and-paraguay).

## Methods

### Sampling area

**Fig 1:**
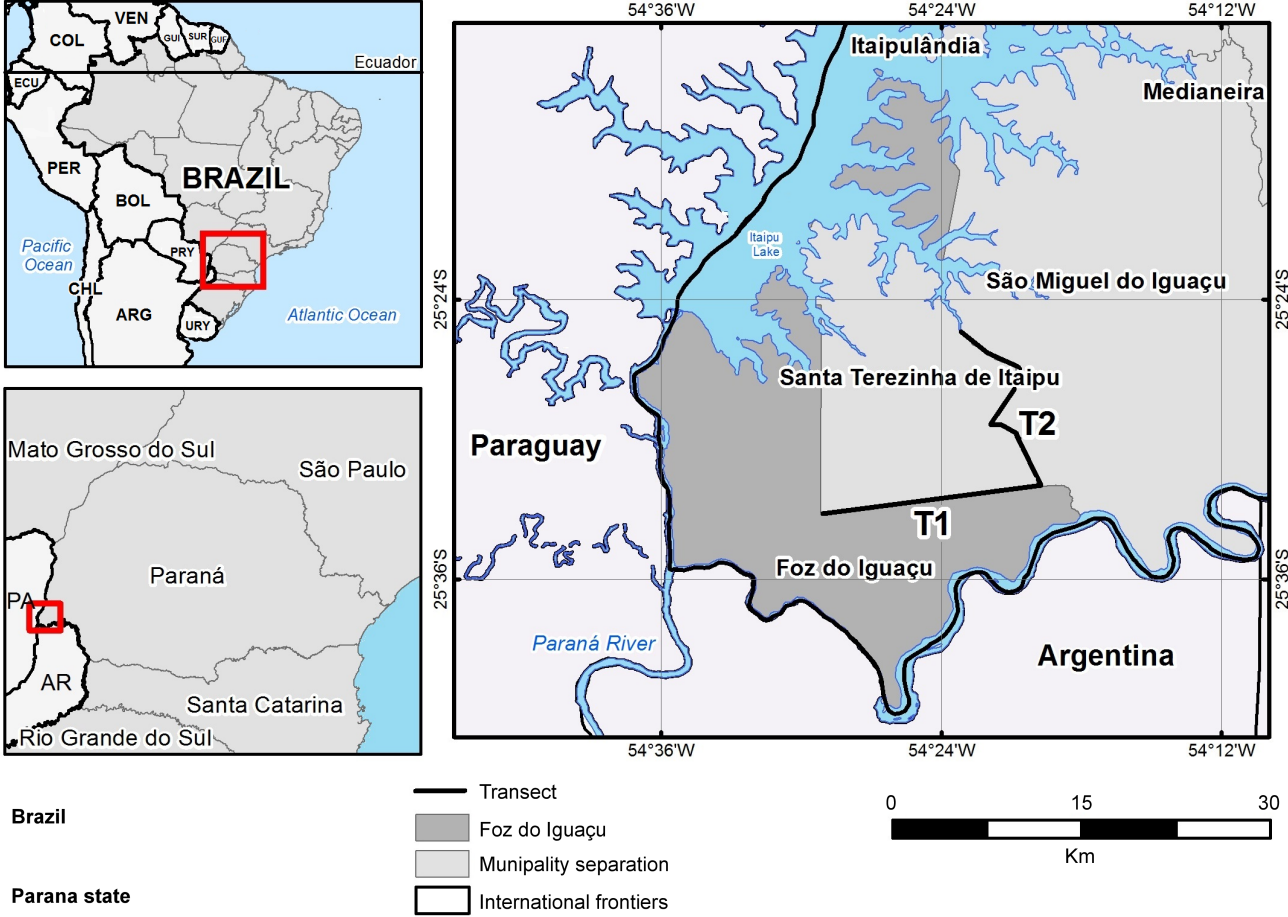
Area where CDC traps were installed for sand fly fauna studies: Foz do Iguaçu city; T1 and T2 transects areas (between Foz do Iguaçu and Santa Terezinha de Itaipu); Santa Terezinha de Itaipu city. Municipal, State and International borders Map was produced with http://www.visualizador.inde.gov.br/.

The survey was undertaken in the extreme western region of Paraná state in southern Brazil, an area known as the triple border with Argentina and Paraguay (Fig 1). The three cities in this area, Ciudad del Este (Paraguay), Puerto Iguazú (Argentina), and Foz do Iguaçu (Brazil), represent an urban area in the Atlantic forest with more than 700,000 inhabitants. We sampled the sand fly populations of the following Brazilian localities: Foz do Iguaçu (FI), Santa Terezinha de Itaipu (STI), and two transects (T1 and T2) through the rural area between these two cities. The number of dogs in FI is about 57,000 (according to the Zoonosis Control Center (CCZ) of Foz do Iguaçu), and about 5,700 in STI (data obtained from Sanitaire Vigilance). Besides dogs and humans, mice, chickens and feral animals are present in the region, and are potential blood sources.

Foz do Iguaçu municipality (25° 32' 52" S-54° 35' 17" W) has an area of 615.02 km^2^ and 256,088 inhabitants—a population density of 416.38 inhabitants/ km^2^ [14]. The region is composed of a conservation area (22% of its area), a rural zone (22%), the Itaipu Lake (24%) and urban areas (32%). Santa Terezinha de Itaipu (25° 21' 44" S-54° 29' 17" W) has 22.783 inhabitants and a density of 80.35 inhabitants/km^2^. The economy is based essentially on livestock, soybean and corn cultivation. A considerable part of the area is delimited by Itaipu Lake with areas of remnant forest. The transects were drawn between FI and STI, this area consists of a rural road that connects FI to STI and passes near to the Iguaçu National Park.

### Entomological sampling

Sand flies were collected between the 25^th^ and 27^th^ of October 2014 in FI, 21^st^ and 24^th^ November 2014 along the transects, and 19^th^ and 21^th^ October 2015 in STI using automatic CDC-Light traps set at 1.5 m above ground level between approximately 6:30 p.m. and 7:30 a.m., during three consecutive rainless nights. Global Positioning System (eTrex10) registered the coordinates of each trap.

For collections in the FI and STI urban areas the cities were divided into a grid of 400 m^2^ (patch) squares (for methodology see [15]). The FI presented 875 patches of 400 m^2^ along four areas of the city (A, B, C and D). The A area corresponds to the administrative area of the city, with the highest density of buildings and near the Paraná River. It contains two forest remnants that occupy a large part of the locality. The B area is a residential region east of A and west of the rural areas. The density of the dwellings is medium and they were distributed almost evenly. The C area is located in the north of the city, bounded by regions A and B in the south, by the Paraná River in the north and west, and by rural areas in the east. The C area is the largest area and is characterized by discontinuous dwellings interrupted by large "green" spaces (sports fields, small cultivated areas, etc.). The D area is located south of the city and regions A and B, is bordered by the Iguaçu (south) and Paraná (west) rivers. It has a relatively high density of buildings, with forest remnants that surround the banks of the rivers.

Due to the limitation of number of available CDC-LT traps, 123 patches from FI (26–28 in the areas A, B, and D, and 42 in the area C, largest area) were selected for the sampling according to the worst scenario criterion [15]. The “worst scenario” is a functional definition to denote a site within the study patch with the greatest probability of sand fly presence due to habitat conditions. These sites presents dense vegetation which provides shadow, humidity and detritus, soil rich in organic material and access to blood sources without the interference of external light. Minimum and maximum distances between traps settled in different patches were 145 and 475 m, respectively.

**Fig 2:**
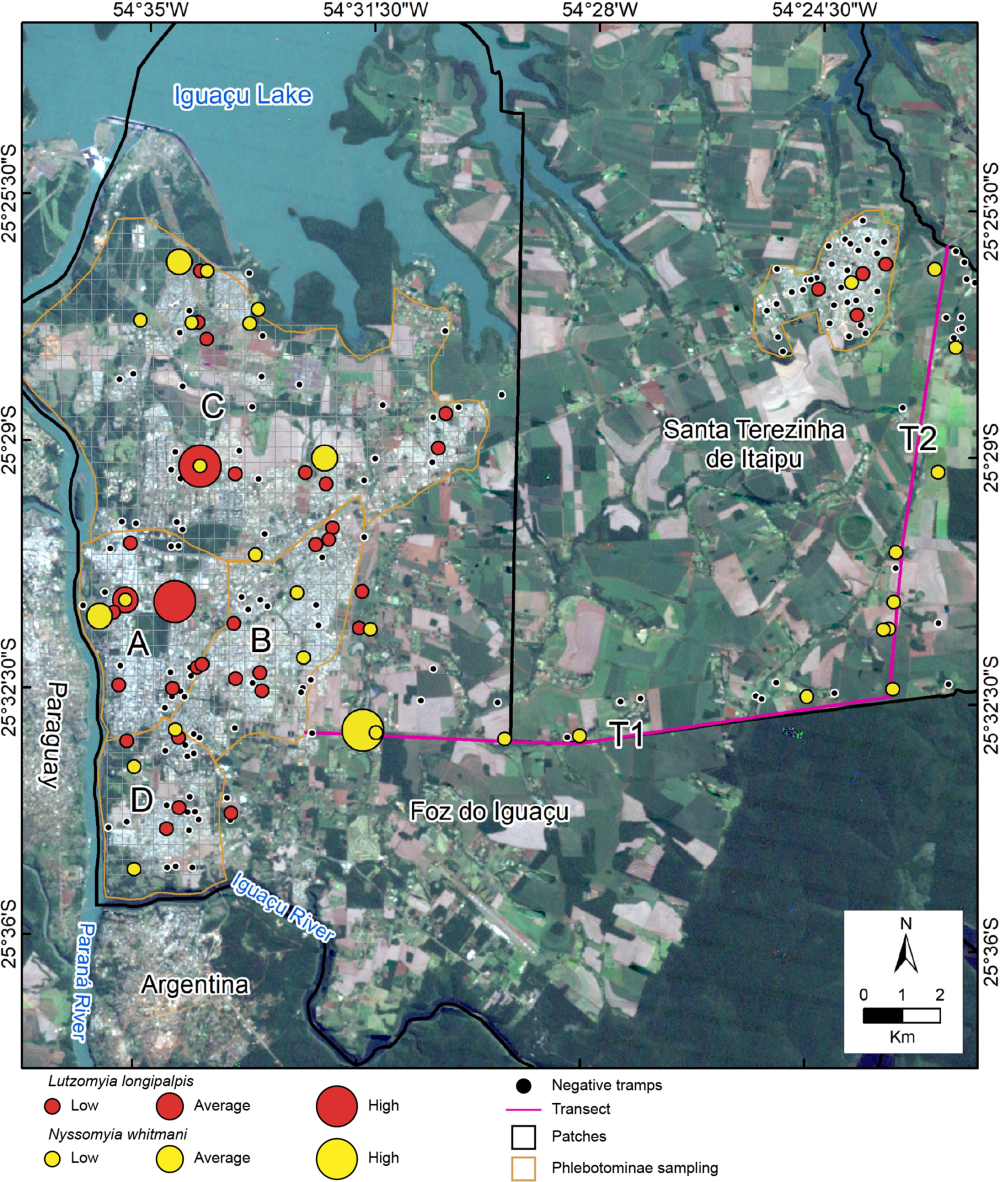
Phlebotominae sand fly distribution in the three survey areas (Foz do Iguaçu, Santa Terezinha de Itaipu and transects). Satellite image free downloaded from https://landsatlook.usgs.gov/.

Sand fly collections were performed in the urban and ruro-urban areas (Fig 2) of STI. The city was also divided into a grid of 400 m^2^ patches and 33 patches were selected for the sampling according to the worst scenario.

Between FI and STI, sand flies were collected along the two transects (T1 and T2) (Figs 1 and 2), that consisted of two sectors in the ruro-urban areas of FI (T1 = 17 km) and the rural area of STI (T2 = 17.5 km). Forty CDC-LTs were distributed along the transects, 20 in each sector (T1 + T2), at intervals of approximately 860 m. Four traps were also installed in the Iguaçu National Park in sites with extensive vegetation cover and in the Itaipu Biological Sanctuary.

To collect sand flies, all the owners/residents that collaborated in the study were informed about the practices and signed the informed consent form.

### Sand fly morphological identification

In the laboratory, the insects were anesthetized with chloroform and separated by sex. The males were stored at 4^o^ C until morphological identification. The individuals were identified according to the morphological taxonomic keys proposed by Galati [16].

Each female was placed on sterile slides containing one drop of sterile saline solution (0.9%). Firstly, the head was sectioned with the aid of two sterile needles. Then, two needles were inserted in the individual, the first through the thorax and the second through the last two abdominal segments. A first linear and slow traction of the needles was performed to detach the last two segments, the spermathecae and ovarian ducts from the thorax. A second slow traction was performed to remove the digestive tract from the thorax. The cut of the anal duct allowed the complete separation of the digestive tract from the last two fragments. The female’s intestine was carried to a microtube with in ethanol 70% for posterior molecular assessing of the presence and the identification of *Leishmania* species. The remaining female (head, thorax, first abdominal segments, spermathecae, ovarian ducts) and the males (kept whole) were clarified using 10% KOH for the visualization of the internal structures as recommended by Rioux et al. [17].

*Evandromyia cortelezzii* and *Evandromyia sallesi* females cannot be distinguished by their morphology, thereby they were named as *Evandromyia* sp. The generic abbreviations of the species proposed by Marcondes [18] were used.

### Molecular identification of the parasites

Pools of intestines (two or three females) from the same trap were extract for assessing the presence of parasites’ DNA. The intestines were macerated in the microtube using a plastic pestle, with 497 μL of lysis buffer (100 mM Tris-HCl, 100 mM NaCl, 25 mM EDTA, 0.5% SDS, pH 8.0). After incubating for 1 h at 55° C with 2% of proteinase K (10 mg/mL solution), the DNA was precipitated using the phenol-chloroform method [19]. The DNA pellet was resuspended in 20 μL of TE-buffer (10 mM Tris-HCl pH 8.0, 1 mM EDTA) and stored at -20° C.

A subset of females (about 70%, see results for details) of all positive traps was selected for *Leishmania* detection according to Schönian et al.´s [20] protocol. The PCR assays were carried out with 5 μL of DNA template of the female pools in a final reaction volume of 25 μL. The reaction contained 1 X PCR buffer (100 Mm Tris-HCl pH 8.0, 0.1 mM EDTA, 1 mM DTT, 50% (v/v) glycerol) (Invitrogen), 2.5 mM MgCl_2_ (Invitrogen), DMSO 2.5% (Sigma), 200 μM of each dNTP (Invitrogen), 0.5 μM LITSR forward primer, 0.5 μM L5.8S reverse primer and 1.4 U of Taq polymerase (Invitrogen). The PCR cycle employed an initial denaturalization step of 94° C for 4 min, followed by 40 cycles of a denaturalization step of 94° C for 30 sec, a hybridization step of 56° C for 30 sec and an extension step of 72° C for 30 sec, and a final extension step of 72° C for 10 min. The cycle procedure was performed in a thermocycler biocycler, MJ96 (Biosystems). DNA of *L*. *infantum* reference strain was used as positive control, and water as the negative control in each reaction. In addition, a 220 bp fragment of the constitutive gene IVS6 (cacophony) of sand flies was used as internal control [21]. All PCR products were assessed through 1.6% agarose gel electrophoresis (1 h at 5 V/cm), stained with ethidium bromide and visualized under ultraviolet light.

The parasite identification was done by performing a RFLP analysis of the amplified ITS1 fragment as proposed by Schonian et al. [20]. The PCR products (10–15 μL) were digested with *Hae*III accordingly, using conditions recommended by the supplier (Hybaid GmbH Heidelberg, Germany). The restriction fragments were submitted to electrophoresis in 2% metaphor agarose (FMC BioProducts Rockland, ME, USA) at 100 V in 0.5× TBE buffer and visualized under ultraviolet light after staining for 15 min in ethidium bromide (0.5 μg/mL). The electrophoresis pattern was compared with the reference strains (i.e., the main *Leishmania* species that cause CL and VL in the Brazil: *L*. *infantum*, *L*. *braziliensis*, *L*. *amazonensis*).

Positive PCR-RFLP products with electrophoresis pattern different from the reference strains were commercially sequenced by the Macrogen Inc. (Seoul, South Korea) to confirm the identification. The electropherograms were manually checked, and the sequences were edited and assembled using BioEdit software and aligned with Guidance 2.0 [22]. The sequences were deposited in the GenBank (accession numbers MG136689 to MG136700).

The identification of the sequenced *Leishmania* samples was obtained from a Bayesian tree constructed using Beast 1.8.4 [23]. Three runs of 30 million MCMC each (3 million of burn-in) were performed with the substitution model (Hasegawa-Kishino-Yano, HKY [24]) defined by Modeltest 2.1.10 [25].

### Host-blood feeding

The blood meal of engorged female sand flies was identified using PCR based analysis on the cytochrome B gene. DNA was extracted from the intestine and its content using the phenol chloroform protocol [19]. The amplifications followed González et al. ‘s [26] protocol. Sequencing was performed commercially by Macrogen Inc. (Seoul, South Korea). The electropherograms were checked manually and the sequences were edited and assembled using BioEdit software. The blood meals were identified using the nucleotide BLAST (Basic Local Alignment Search) tool on the GenBank.

### Micro, meso and macro scale environmental variables

To assess the environmental variables that affect the presence and abundance of sand fly species, micro and meso scale factors were evaluated. Furthermore, interviews were conducted, by domicile, during the sand fly collection period. Several questions were recorded and environmental variables were measured. Twenty-nine variables were selected for this study (Table 1), including mean of the minimum and maximum temperatures, and relative humidity of each night.

**Table 1:** Survey of environmental variables in meso and micro-scale and sand fly food source and their contribution (assessed using maximum entropy approach) to the presence of *Lutzomyia longipalpis* and *Nyssomyia whitmani* in the Brazilian side of the triple border.

Group	Variable	Contributions (%)	
		*Lu*. *longipalpis*	*Ny*. *whitmani*
**Site**	Foz do Iguaçu	*	*
	Transect	*	*
	Santa Terezinha de Itaipu	*	*
**Public services**	Garbage collection	6	0
	Street lighting	0	0
**Mesoescale**	Trees 250 m	7	7
	Herbaceous 250 m	11	10
	Soil 250 m	1	2
	Urban 250 m	14	0
	Water 250 m	3	4
	Cultivation 250 m	3	9
	Heterogeneity 250 m	0	0
**Microescale**	Trees 25 m	4	1
	Herbaceous 25 m	0	1
	Soil 25 m	2	6
	Urban 25 m	3	0
	Water 25 m	1	3
	Cultivation 25 m	1	1
	Heterogeneity 25 m	0	0
	Fallen leaves and fruits	0	0
	Insecticide	3	0
	Garbage	0	0
**Temperature (°C)**	Minimum temperature	13	5
	Maximum temperature	8	0
	Humidity	6	27
**Food Supply**	Plenty of dogs	8	2
	Presence of rodents	1	4
	Presence of chicks	3	12
	Plenty of chickens	0	3
**Altitude**	Altitude	0	2
**Waters bodies and rivers**	Distance from water bodies	1	2

The Normalized Difference Vegetation Index (NDVI) was used to highlight the presence of vegetation in each studied area and the plant biomass [27]. Maps were constructed based on bands 6 (B6 –near infrared band) and 5 (B5 –near red band, respectively) of the LandSat 8 satellite. The months with largest volumes of precipitation are in March and the smallest precipitate volumes in August [28]. To generate the NDVI, three LandSat 8 satellite images were used from February, August, and October 2014 for FI and for transects. For STI the analyses, satellite images was acquired from the same months in 2015. In the Geographic Information Systems (GIS) environment, the following formula was applied using bands 6 and 5: NDVI = [(B6 –B5)/(B6 +B5)]. The Normalized Difference Vegetation Index present values ranging from -1 to +1. Values closer to +1 indicate a high presence of vegetation, and values closer to -1 represent absence of vegetation. The maps of forest fragments (vegetal remnant) were generated from the data of the Environment Ministry´s project for managing the Biodiversity of the Paraná River´s corridor [29]. Hypsometry and altimetry maps were generated based on the Shuttle Radar Topography Mission (SRTM) and Digital Terrain Model (DTM), with spatial resolution of 30 m in the X band. The DTM was purchased from the United States Geological Survey (USGS) website. The maps with the hydrographic network were generated from the official database of the water bodies’ network of the State of Paraná in the scale 1:50,000, in collaboration with several public institutions (e.g., SANEPAR, COMEC, ITAIPU, ITCG, Instituto das Águas do Paraná, IAP). The average annual and seasonal air temperature data were acquired based on Paula [30], which generated a serial historical average (~30 years).

The maps were processed using the open software QGIS version 2.18.

### Statistical analyses

The relative abundance of the vectors was calculated as: number of *Lu*. *longipalpis* by peridomicile/total domicile surveyed [31].

Path Analyses were implemented with the package 'plspm' 0.4.7 [32] in the R 3.3.3 [33] for assessing the influence of each environmental variable (Table 1) on sand fly abundance. In this analysis, the variables were grouped at mesoscale (proportion in a plot of 250 m of land with trees, herbaceous, soil, urban, water and crops: heterogeneity), microscale (proportion in a 25 m plot of land of trees, herbaceous, soil, urban, water and crops, presence of garbage and fallen leaves and fruits: heterogeneity), animal supply (presence of chicks and rodents, and abundance of dogs and chickens), public services (lighting and garbage collection), temperature and humidity (humidity, minimum and maximum temperatures), and study areas (FI, Transect and STI). The heterogeneity is the number of different ground cover (i.e., trees, herbaceous, soil, urban, water and crops) in the area (25 and 250 m) of each trap. Altitude, distance from the water and abundances of *Lu*. *longipalpis* and *Ny*. *whitmani*, were kept isolated. The influence of the environmental variables was calculated only to these two species because only they presented sufficient number of specimens’ (n > 200, see results for details). The influence of sand fly species abundance on other sand fly species was not tested since their abundances hypothetically do not affect directly the abundances of other species, and their collinearity can be explained due to the sharing of ecological characteristics (phylogenetic conservatism).

Additionally, the percentage of contribution of each variable on the presence of each species, in each trap, was assessed with a maximum entropy approach in the Maxent 3.4.1 [34, 35]. The validation of the model was assessed using the area under (AUC) the receiver operating characteristic (ROC) curve.

### Accession numbers

**Fig 3:**
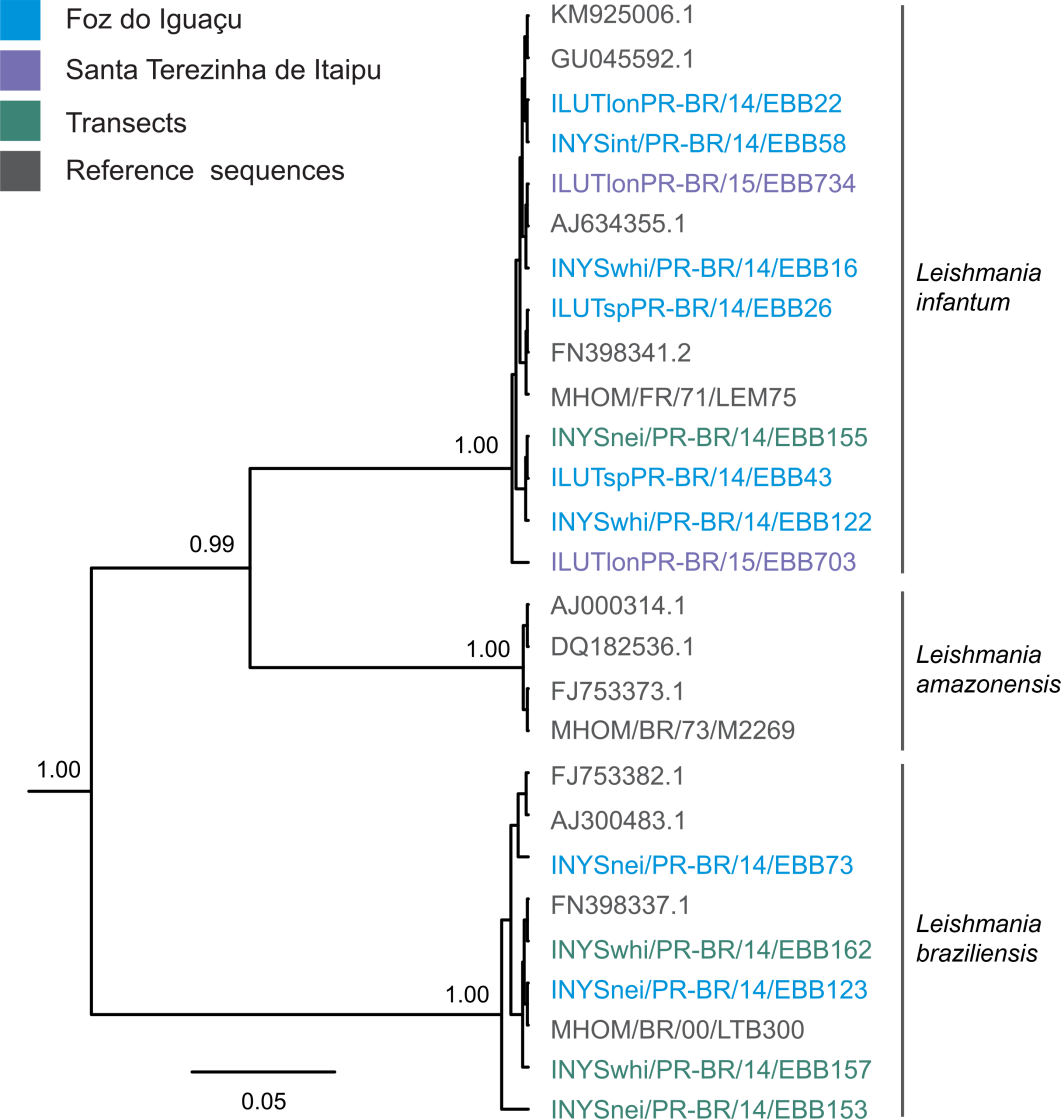
Bayesian tree constructed for the identification of *Leishmania* found in sand flies from Foz do Iguaçu, Santa Terezinha de Itaipu and transects. Only posterior probabilities higher than 0.95 are presented.

All sequences obtained in the present work, as well as sequence of the reference strain of *L*. *infantum* (MHOM/FR/78/LEM 75), *L*. *braziliensis* (MHOM/BR/75/M2903), and *L*. *amazonensis* (MHOM/BR/73/M2269) were used in the Bayesian tree construction. In addition the sequences of *L*. *infantum* (AJ634355.1, FN398341, GU045592, KM925006), *L*. *braziliensis* (AJ300483, FJ753382, FN398337) and *L*. *amazonensis* (AJ000314, DQ182536, FJ753373) deposed in the GenBank were added for the tree construction. Our isolates regrouped with *L*. *infantum* and *L*. *braziliensi*s (Fig 3).

## Results

### Prospection of vectors

A total of 1,202 sand fly specimens were collected, 91.3% (1,097) of them in FI, 7.7% (93) along the two transects and 1% (12) in STI city (Tab 2). Sand flies were captured in 81 of 196 (41.3%) patches. The predominant species was *Lu*. *longipalpis* (in 41 traps, 21%). *Nyssomyia whitmani* was present in 34 traps (17%), and *Ny*. *neivai* in 20 (10%).

A total of 1,097 sand flies, belonging to seven species, were captured in 44.7% of the FI´s 123 traps. *Lutzomyia longipalpis* was the most abundant species (55.7%) in the three habitats (rural, ruro-urban and urban) of the municipality’s regions (A–D) (Table 2). *Nyssomyia whitmani* was present predominantly in sites close to forest remnant. In the two transects (T1 + T2), the most abundant species were *Ny*. *whitmani* (50.0%), *Mg*. *migonei* (19.4%) and *Ny*. *neivai* (18.3%). *Nyssomyia whitmani* was dominant in both T1 and T2 regions (39.3 and 67.6%, respectively), followed by *Ny*. *neivai* (17.9 and 19.9%, respectively). *Migonemyia migonei* was present in the T1 (30.3%) and T2 (2.7%), *Lu longipalpis* was not found in the transect captures (Table 3). In STI, sand flies were found in eight of the 33 traps surveyed (Table 2). *Lutzomyia longipalpis* was present in four quadrants (7, 8, 15 and 18), *Ny*. *whitmani* in three quadrants (4, 22 and 26) in the ruro-urban region, and *Ny*. *neivai* in 14 CDC-LT.

**Table 2:** Number of traps (Nt) examined, and number (N) and percentage (within parentheses) of positive traps for *Lutzomyia longipalpis* and *Nyssomyia whitmani* in three habitats (rural, ruro-urban and urban) in Foz do Iguaçu, Santa Terezinha de Itaipu and two transects between these cities.

Strata	Foz do Iguaçu			Transects			Santa Terezinha de Itaipu		
	Nt	*Lu longipalpis*	*Ny whitmani*	Nt	*Lu longipalpis*	*Ny whitmani*	Nt	*Lu longipalpis*	*Ny whitmani*
**Rural**	3	1 (33.3)	1 (33.3)	35	0 (0.0)	11 (31.4)	0	0 (0.0)	0 (0.0)
**Ruro-urban**	26	10 (38.4)	5 (19.2)	5	3 (60.0)	3 (60.0)	3	1 (33.3)	0 (0.0)
**Urban**	94	26 (28.7)	2 (15.9)	0*	*	0 (0.0)	30	3 (10.0)	1 (3.3)
**Total**	123	37 (28.9)	21 (16.4)	40	3 (7.5)	14 (35.0)	33	4 (12.1)	1 (3.0)

*Transects present no urban area, only small community.

**Table 3:** Number of positive traps (Nt) and number of sampled sand flies (Nsf) in each area and of each sand fly species. The number inside the parenthesis represents the percentage of positive traps and the percentage of each species in each area. A total of 123 traps in Foz do Iguaçu, 40 traps in the transect and 33 traps in the Santa Terezinha de Itaipu was assessed.

Species	Foz do Iguaçu		Transects		Santa Terezinha de Itaipu	
	Nt	Nsf	Nt	Nsf	Nt	Nsf
***Lu*. *longipalpis***	37 (28.9)	611 (55.7)	0 (0.0)	0 (0.0)	4 (12.1)	8 (66.7)
***Mg*. *migonei***	0 (0.0)	0 (0.0)	3 (15.0)	18 (19.4)	0 (0.0)	0 (0.0)
***Ny*. *neivai***	12 (9.4)	128 (11.6)	7 (35.0)	17 (18.3)	3 (9.0)	3 (25.0)
***Ny*. *whitmani***	21 (16.4)	309 (28.3)	10 (50.0)	47 (50.5)	1 (3.0)	1 (8.3)
***Brumptomyia* sp**	3 (2.3)	19 (1.7)	3 (15.0)	7 (7.5)	0 (0.0)	0 (0.0)
***Pa*. *shannoni***	1 (0.8)	3 (0.4)	3 (15.0)	3 (3.2)	0 (0.0)	0 (0.0)
***Pi*. *monticola***	0 (0.0)	0 (0.0)	1 (5.0)	0 (0.0)	0 (0.0)	0 (0.0)
***Pi*. *pessoai***	0 (0.0)	0 (0.0)	1 (5.0)	0 (0.0)	0 (0.0)	0 (0.0)
***Mi*. *quinquefer***	6 (4.7)	15 (1.4)	0 (0.0)	0 (0.0)	0 (0.0)	0 (0.0)
***Evandromyia* sp**	1 (0.8)	2 (0.2)	0 (0.0)	0 (0.0)	0 (0.0)	0 (0.0)
***Pi*. *fischeri***	0 (0.0)	0 (0.0)	1 (5.0)	1 (1.1)	0 (0.0)	0 (0.0)
**Without identification**	5 (3.9)	10 (0.9)	0 (0.0)	0 (0.0)	0 (0.0)	0 (0.0)
**Total**	**55 (44.7)**	**1097**	**18** **(45.0)**	**93**	**8** **(24.2)**	**12**

The greatest relative abundances of *Lu*. *longipalpis* were in zones A (9.76) and C (7.02) in Foz do Iguaçu, and the lowest was in STI (0.18). The greatest number of *Ny*. *whitmani* was recorded in the D zone in FI, followed by transect zones (2.35) (Table 4). The male to female ratio for *Lu*. *longipalpis* was 4.64:1 in FI and 0.75:1 in STI. For *Ny*. *whitmani*, sexual ratio was 1.80:1 in FI, 3.18:1 in the transect area and 0.67:1 in STI urban area (Fig 2).

**Table 4:** Relative abundance of the predominant species in the Foz do Iguaçu, Transects and Santa Terezinha de Itaipu (STI).

Foz do Iguaçu/area	*Lu*. *longipalpis*	*Ny*. *whitmani*	*Ny*. *neivai*	*Mg*. *migonei*
**A**	9.76	1.11	0.19	0.00
**B**	1.15	0.57	0.00	0.00
**C**	7.02	2.02	0.52	0.00
**D**	0.61	8.00	0.34	0.00
**Total FI**	4.37	1.14	0.30	0.00
**T1**	0.00	1.10	0.50	0.85
**T2**	0.00	1.25	0.35	0.05
**STI**	0.18	0.15	0.03	0.00

### *Leishmania* infection rate in sand fly females and identification of parasites by molecular biology

PCR-RFLP technique was applied on 179 of the 264 female sand flies collected in FI to identify the species of *Leishmania* that infect them. Among the 37 traps where female sand flies were collected, 12 (32.4%) were responsible for the 64 infected females (35.8% of the total of females assessed) (Table 5). A great part of these infections was found in four traps (43.1% in the trap 321; 21.5% in the trap 329; 9.8% in the traps 448 and 470).

**Table 5:** Number of females assessed and of positive PCR-RFLP for *Leishmania* species, and the identification of the *Leishmania* species in sand flies collected in Foz do Iguaçu, along the transects (T1 and T2), and Santa Terezinha de Itaipu.

FOZ DO IGUAÇU		positive	Parasite identified
Species	Female assessed	PCR-RFLP	
*Lu longipalpis*	56	41	*L*. *infantum*
*Ny whitmani*	40	5	*L*. *infantum*
			*L*. *braziliensis*
*Ny neivai*	7	2	*L*. *infantum*
			*L*. *braziliensis*
*Lu quinquefer*	9	1	
*Lutzomyia* sp	34	8	*L*. *infantum*
**TRANSECT 1 + 2**			
**Species**			
*Ny whitmani*	13	5	*L*. *braziliensis*
*Ny neivai*	8	2	*L*. *braziliensis*
*Lutzomyia* sp	8	0	
**SANTA TEREZINHA DE ITAIPU**			
**Species**			
*Lu longipalpis*	4	0	
**TOTAL**	**179**	**64**	

The sequences of the 11 sand flies captured in FI obtained from the PCR-RFLP supported that two *Lutzomyia* sp., a *Ny*. *neivai* and two *Ny*. *whitmani* were positive for *L. infantum*, and a *Ny*. *neivai* and a *Ny*. *whitmani* were positive for *L. braziliensis* (Table 5). Two *Ny*. *whitmani* and a *Ny*. *neivai*, from the transects were positive for *L. braziliensis*.

### Host-blood feeding

Sixteen engorged *Lu*. *longipalpis* females were collected and their blood meal identified. The blood belonged vertebrate species (Table 6): *Canis familiaris* (6 females– 37.5%), *Mus musculus* (5 females– 31.3%), *Homo sapiens* (4 females– 25.0%) and *Dasypus novemcinctus* (1 female– 6.2%).

**Table 6:** Identification of the blood meal sources in 16 females *Lutzomyia longipalpis* captured in the Brazilian side of the triple border.

Species	Common name	Number of females	%	Sequences similarity	Blast access number
***Homo sapiens***	Human	4	25.0	99	AY509658
***Canis familiaris***	Dog	6	37.5	99	DQ309764
***Dasypus novemcinctus***	Nine-banded Armadillo	1	6.2	89	AF493839.1
***Mus musculus***	House Mouse	5	31.3	93	AB819920.1

### Spatial distribution of the sand flies

To understand the spatial distribution of the sand fly species, the most abundant species were chosen and separated by area (Fig 2). For FI, *Lu*. *longipalpis* was present in the four sampled areas, especially in the central (region A) and northwest (region C) areas. *Nyssomyia whitmani* was present especially in ruro-urban areas and in forest remnant. In the rural (T1 + T2) areas, *Ny*. *whitmani* and *Ny*. *neivai* were sympatric. The greatest abundance was in the forest remnant and peridomicile areas in the Iguaçu National Park. In STI, the spatial distribution of the vectors indicated that sand fly fauna was greater near to the federal highway BR 277, which crosses this city.

### Abundance and spatial distribution of sand fly populations and environmental variables

The study of the environmental variables as hydrography, forest remnant, hypsometry, NDVI, and temperature were performed only for the most abundant species (i.e., *Lu*. *longipalpis*, *Ny*. *whitmani*).

**Fig 4:**
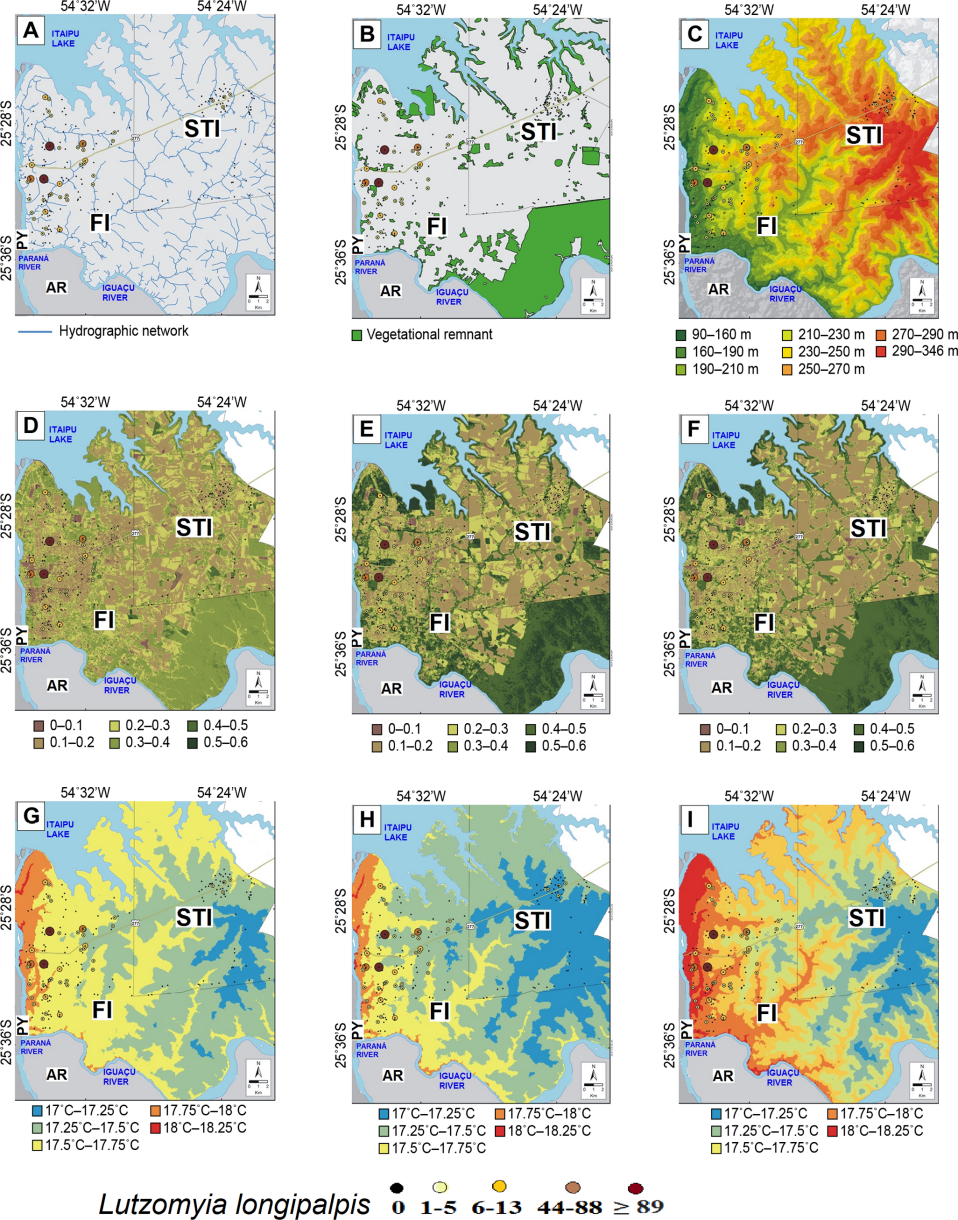
**Abundance and spatial *Lutzomyia longipalpis* distribution showing the number of collected specimens: (A) hydrographic network, (B) forest remnant, (C) hypsometry in m, (D) normalized vegetation index—winter period, (E) normalized vegetation index summer period, (F) normalized vegetation index—spring period, (G) winter temperatures, (H) summer temperature, (I) average temperature over the last 30 years in Foz of Iguaçu (FI) and transects areas (T1 + T2) October/November 2014 and Santa Terezinha de Itaipu (STI) October 2015.** The following layers were used for the maps building: 1) Hydrographic network from Institute of the Waters of Parana available in http://www.aguasparana.pr.gov.br/; 2) Remaining fragments of vegetation, NDVI and Digital terrain model in Landsat 8 sensor. Image courtesy of the U.S. Geological Survey https://lta.cr.usgs.gov/citation/ and https://earthexplorer.usgs.gov/. Legends: AR–Argentine, PY- Paraguay.

**Fig 5:**
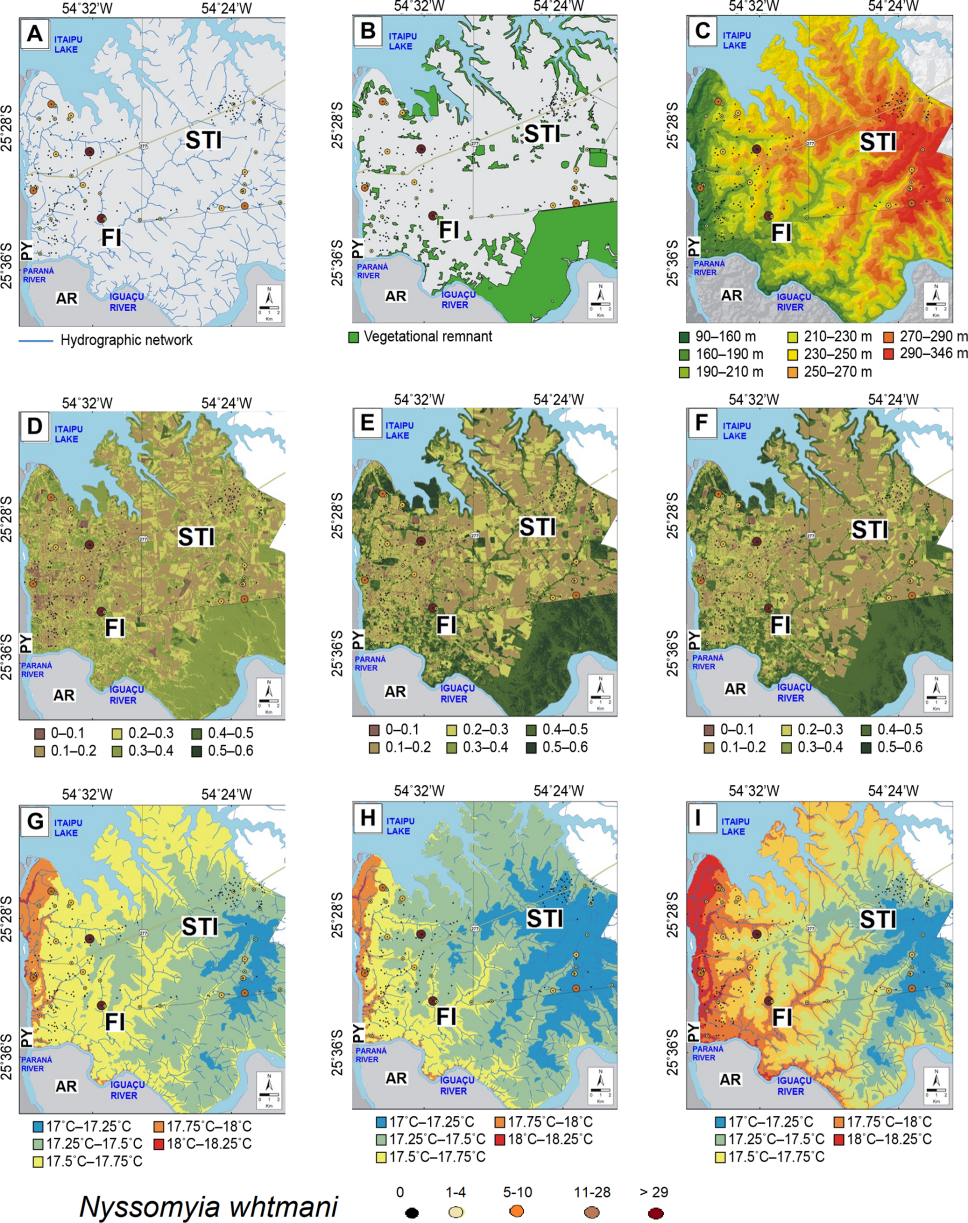
**Abundance and spatial *Nyssomyia whitmani* distribution showing the number of collected specimens: (A) hydrographic network, (B) forest remnant, (C) hypsometry in m: (D) normalized vegetation index—winter period, (E) normalized vegetation index summer period, (F) normalized vegetation index spring period, (G) winter temperatures, (H) summer temperature, (I) average temperature over the last 30 years in Foz do Iguaçu (FI) and transect areas (T1 + T2) October/November 2014 and Santa Terezinha de Itaipu (STI) October 2015.** The following layers were used for the maps building: 1) Hydrographic network from Institute of the Waters of Parana available in http://www.aguasparana.pr.gov.br/; 2) Remaining fragments of vegetation, NDVI and Digital terrain model in Landsat 8 sensor. Image courtesy of the U.S. Geological Survey https://lta.cr.usgs.gov/citation/ and https://earthexplorer.usgs.gov/. Legends: AR–Argentine, PY- Paraguay.

*Lutzomyia longipalpis* and *Ny*. *whitmani* (Figs 4 and 5, respectively) were present in the areas close to water sources. This variable influences the microclimate of the region, especially humidity, precipitation and wind flows. In STI, sand flies were recorded both close to and distant from water bodies. However, similar to FI, STI is also influenced by the Itaipu reservoir, rivers and other aquatic environs. Although rivers are abundant in the transects, *Lu*. *longipalpis* was not found.

The distribution of the vectors showed that *Ny*. *whitmani* was more abundant near the forest remnants and in the rural zone, including the National Park. On the other hand, *Lu*. *longipalpis* was present in urban areas irrespective to the presence of vegetation. In STI, *Lu*. *longipalpis* was captured in the downtown zone (Figs 4 and 5). It was present in areas whose altitude varied from 90 to 270 m above sea level (masl), while in the STI the population was present in higher altitudes, between 250 to 290 masl. *Nyssomyia whitmani* was present between 90 and 346 masl in both FI and STI. The vegetation index (NDVI) indicated that *Lu*. *longipalpis* and *Ny*. *whitmani* were influenced by the forest corridors within the city of FI (Figs 4 and 5). *Nyssomyia whitmani* was positively influenced by the remnant forest in Iguaçu National Park, an Integral Protection Conservation Unit. The presence of forest remnant affected more *Ny*. *whitmani* than *Lu*. *longipalpis*. The greatest abundance was always near to areas with abundant vegetation.

During the sand fly collection period (three consecutive night), in FI, the average minimum temperatures registered were 19.0, 22.3 and 21.8° C for each night, while maximum temperatures were 27.4, 33.2 and 33.3° C, respectively. The average temperature for the last 30 years in FI, where *Lu*. *longipalpis* was recorded, ranged from 17.5 to 18.0° C in the winter, and from 25.5 to 26.2° C in the summer, and the annual temperature ranged from 21.8 to 22.5° C. In STI, the minimum temperature recorded in our sampling was 25.4° C and the maximum was 36.4° C. The humidity varied from 71 to 75%. In the winter, the temperature of this region ranged from 17.2 to 18.0° C, and from 25.2 to 26.2° C in summer.

### Variables influencing the abundance of vectors

The Path modeling performed to test the influence of groups of variables on the sand fly abundance (*Lu*. *longipalpis*, *Ny*. *whitmani*) indicated that only the animal food supply group (loadings: dog abundance = 0.68, presence of rodents = 0.02, presence of chicks = 0.52, abundance of chickens = 0.50) significantly affects the abundances of *Lu*. *longipalpis* (Path coefficient of 0.33, *p* < 0.01, R^2^ of the predictor model = 17%). The maximum entropy approach (AUC = 0.834, Table 1) supported that urban 250 m (14%), minimum temperature (13%), herbaceous 250 m (11%), maximum temperature and plenty of dogs (8% each) are the main variables that contributes to the presence of *Lu*. *longipalpis* in the region.

**Fig 6:**
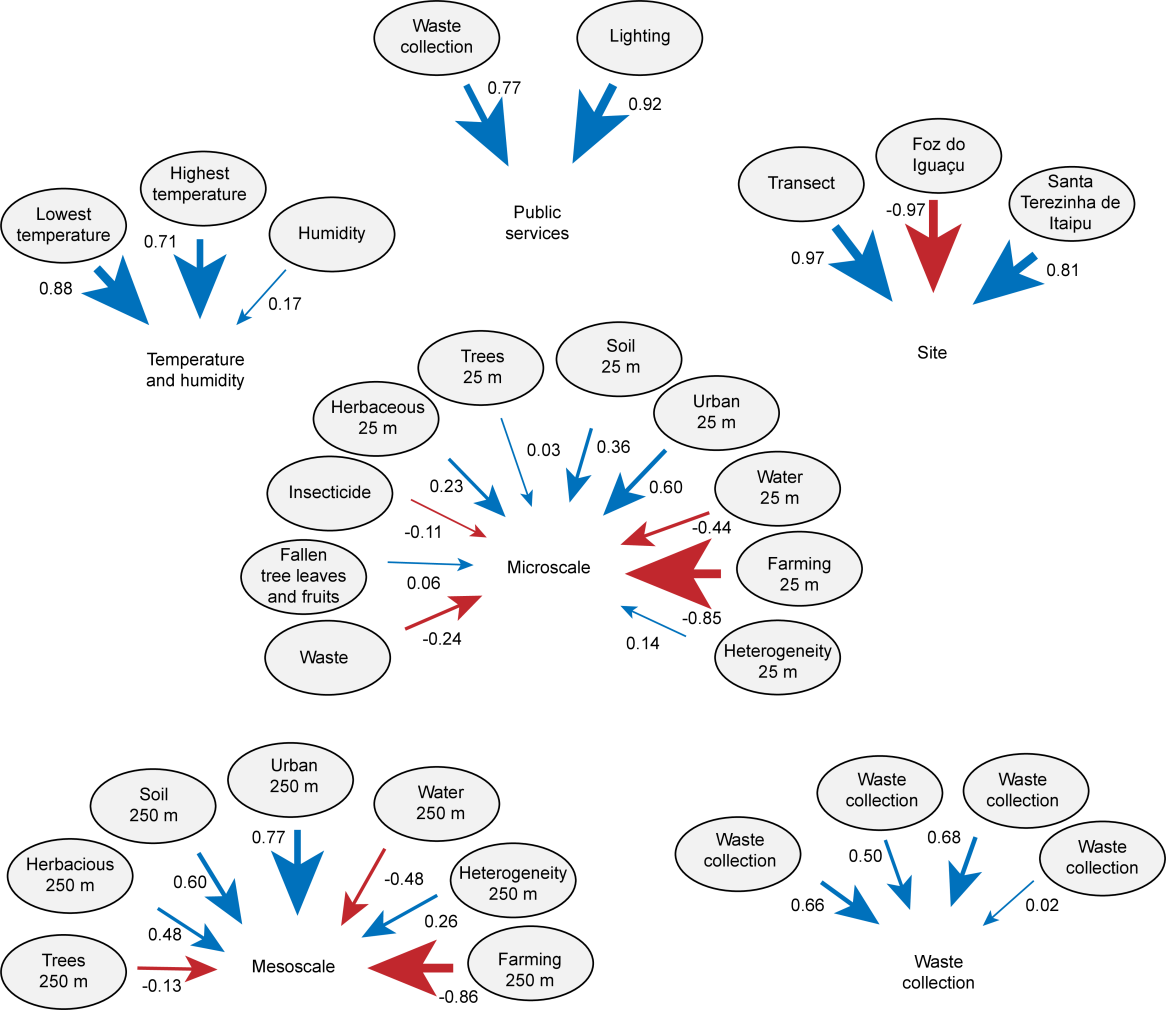
Loadings of the variables public services, temperature and humidity, food supply, site, mesoscale, microscale analyzed in each group.

**Fig 7:**
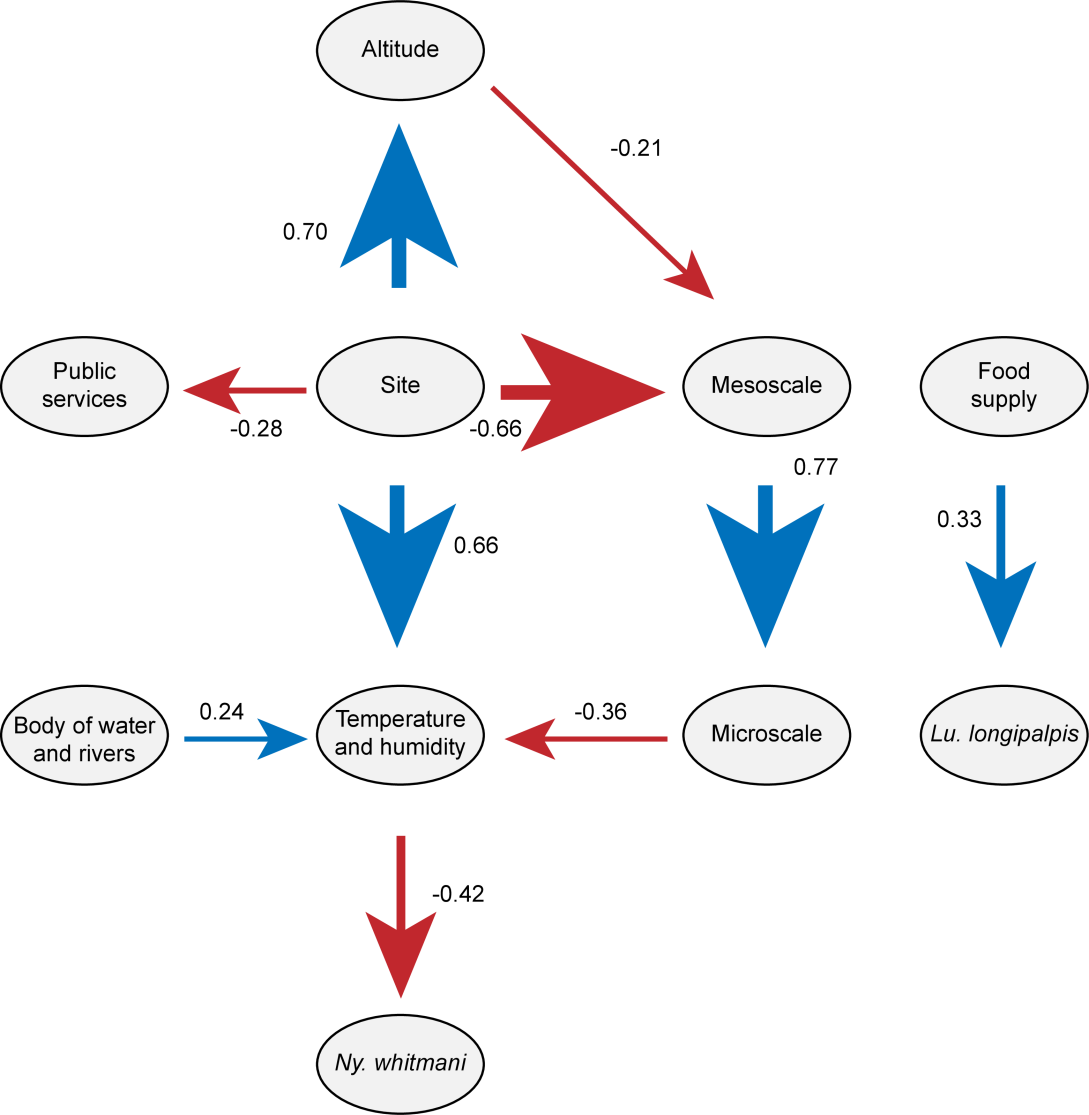
Network of influence of the public services, temperature and humidity, food supply, site, mesoscale, microscale on the abundance of *Nyyssomyia whitmani* and *Lutzomyia longipalpi*s.

The abundance of *Ny*. *whitmani* was only affected by the temperature and humidity (loadings: minimum temperature = 0.88, maximum temperature = 0.71, humidity = 0.17) (Path coefficient of -0.42, *p* < 0.01, R2 of the predictor model = 9%) (Figs 6 and 7). Similarly, the maximum entropy analysis (AUC = 0.891, Table 1) supported that the humidity (27%) presented the large contribution to the presence of *Ny*. *whitmani*, followed by the presence of chicks (12%) and herbaceous at 250 m (10%). The remaining variables contributed less than 10% to the presence of the species.

## Discussion

A total of 1,202 sand flies, composed of 11 species, were collected on the Brazilian side of the triple border. The dominant species were *Lu*. *longipalpis*, *Ny*. *whitmani* and *Ny*. *neivai* in FI, *Ny*. *whitmani*, *Ny*. *neivai* and *Mg*. *migonei* along the transects, and *Lu*. *longipalpis* and *Ny*. *whitmani* in STI. The most common species, *Lu*. *longipalpis*, is widely dispersed in FI (37/123 sites evaluated), less abundant in STI (4/33 sites evaluated) and absent in the transect areas. Of the environmental variables that we assessed, only the animal food supply, and temperature and humidity affected *Lu*. *longipalpis* and *Ny*. *whitmani* abundance.

We detected *L. infantum* DNA in *Lu*. *longipalpis*, *Ny*. *whitmani* and *Ny*. *neivai*. This represents the first record of this parasite in these species in Paraná state. In a previous study in the border areas of the Paraná state with neighboring countries, Consolim et al. [36] studied six Brazilian municipalities (FI São Miguel do Iguaçu, Santa Helena, Marechal Candido Rondon, Guaíra and Terra Roxa) between 1979 and 1986. *Lutzomyia longipalpis* was not registered among the eight sand fly species that they found (i.e., *Martinsmyia alphabetica*, *Pintomyia patinae*, *P*. *fischeri*, *Ny*. *intermedia* s.l., *Ny*. *whitmani*, *Psathyromyia shannoni*, *Mg*. *migonei*, *Micropygomyia quinquefer*). Similarly, *Lu*. *longipalpis* was not recorded in 37 municipalities of Paraná State between 2004 and 2005 [37]. The first report of this species in the state was done by Santos et al. [10] in 2012 in FI, although the parasite infection was not investigated. Our study supports for the first time that *Lu*. *longipalpis* is widely distributed in the region and is abundant in urban areas of FI (56.8%) and STI (12%). Furthermore, we describe the first record of *Ev*. *edwardsi* and *Micropygomyia ferreirana* in FI as well as vectors capable of transmitting *L. braziliensis* (*Pi*. *pessoai*, *Pi*. *fischeri*, *Ny*. *intermedia*, *Ny*. *neivai* and *Ny*. *whitmani*) in FI, STI and the transects. These species were present mainly in areas with remnant forest and sources of water.

The spatial distribution and the maximum entropy analysis (percentage of urban areas were the main variable that affect the presence of the species) show that the distribution of *Lu*. *longipalpis* is predisposed to ruro-urban regions that combine both urban and rural characteristics, where there are remnants of vegetation, debris and the presence of animals such as rodents, dogs and chickens. In FI, we can clearly see that there is a link between urban and rural areas. However, *Lu*. *longipalpis* was absent in the transects of our cross-sectional study, showing so far that it lacks of adaptation to this previously rural [15] new ecotope where there is a larger rural *Ny*. *whitmani* population that is also present in peri-urban zones. This suggests a potential change in the pattern of cutaneous leishmaniasis transmission.

In Neotropical countries, it is very difficult to distinguish essentially urban areas. Classifications by different governmental bodies use the number of dwellings per hectare and the population size. In the United Kingdom, an area is considered as being rural if the settlements have less than 10,000 inhabitants (https://www.gov.uk/government/statistics/2011-rural-urban-classification). In FI 32% is urban, 22% rural, and the rest is divided between national park area (22%) and the Itaipu Lake (24%) [14]. Thus, some places previously classified as urban areas are transitional areas that can be classified as ruro-urban, maintaining a forest remnant, with potential characteristics for sand fly breeding, such as temperature and humidity. In four of the surveyed units (A–D) in FI, there are rural areas near to the downtown city with *Lu*. *longipalpis*. Also, in neighborhoods bordering Argentina and Paraguay, there are transitional areas between rural and urban, and houses with large backyards, chicken houses and a lot of trees [15]. These conditions appear to be adequate habitats for sand fly reproduction, as observed in the all FI units, where four of the six traps were positive in unit A, two of the 10 in unit C, and two of the six in unit D. These sites were characterized as being ruro-urban.

For the analysis of sand fly spatial distribution in FI, only the two species with the highest abundances (*Ny*. *whitmani* and *Lu*. *longipalpis*) were here considered. In FI, *Ny*. *whitmani* was present near the Itaipu Lake and in humid areas with lower temperatures. The influence of these factors on the distribution of this species was supported by the Path Analysis and maximum entropy analysis (Table 1 and Fig 7). *Nyssomyia whitmani* is present in all areas of FI, including downtown and in the areas with the highest temperature.

The presence of large numbers of *Lu*. *longipalpis* throughout the city combined with the large numbers of infected dogs reveals a scenario of potential autochthonous humans and dog VL according to Thomaz-Soccol et al. [13]. There is a huge risk evidenced by the Path Analysis and maximum entropy analysis that the presence and the number of dogs influence positively the prevalence of *Lu*. *longipalpis*, an effective vector of VL. Once dogs are associated with humans, it maximizes the probability of VL transmission under these conditions. The presence of sand fly females engorged with human and dogs meal suggests that the transmission of VL to humans may became more frequent with the increasing of the infection rate of sand flies and dogs. Moreover, in the extreme west Paraná, climate change, associated with large human population flow, trade-related migration, international tourism (FI is the second tourist destination in Brazil), and unchecked animal transport can allow rapid VL dissemination.

The possibility of transmission of *L*. *infantum*, by other sand fly species besides *Lu*. *longipalpis*, may aggravate this scenario. In our study, we found DNA of *L*. *infantum* in *Ny*. *whitmani* and *Ny*. *neivai*. The presence of *L*. *infantum* in other sand fly species is not a surprise since similar results have been found in other Brazilian regions and the Argentinean border in 15 different sand flies species [38–46] and need to be analyzed from the point of view of the Paradigm of Stockholm [47]. According to this paradigm, the inclusion of new host species (vector and reservoirs) is often associated with inherent or environmental characteristics that promote the opportunity of contact between species of potential associates. Traditional paradigms of co-speciation hypothesized that the co-adaptive responses between parasite and host/vector generates specificity in an association (i.e., parasite *L*. *infantum* and the vector *Lu*. *longipalpis*), and a new and rare developing of new adaptations (novel capacities) can provide host shifts. However, the Stockholm Paradigm of evolution of associations proposed suggests that host switching occurs more frequently than previously thought (e.g., EID–Emergent Infectious Diseases) and, thus, it should be expected that parasites utilize a broader range of host species than traditionally expected. Parasites incorporate new host species through ecological fitting (genetic diversity, phenotypic plasticity and phylogenetic conservationism) [48], without the needed of acquisition of new genetic capacities. The introduction of *L. infantum* in the Neotropical region is clearly an example of ecological fitting. *Leishmania infantum* presents a generalist behaviour around the world, using species of *Phlebotomus* as vector in the Europe, Africa and, Asia (see Ready [49] for details), and *Lu*. *longipalpis* in the Neotropical region [50]. Hence, it should not be surprising that *L*. *infantum* is capable of parasitizing sand fly species other than species of *Phlebotomus* from the Old World, such as *Lu*. *longipalpis* following its introduction to the Neotropical region–both host species likely represent similar vectorial resources for *L*. *infantum*. Accordingly, the Paradigm of Stockholm suggests that the probability of *L*. *infantum* completes its life cycle using other sand fly species than *Lu*. *longipalpis* in the Neotropical region cannot be neglected. The process known as multi-level ecological fitting, Malcicka [48] also predicts that ecological fitting may occur at any level of the life cycle and often host species are substituted simultaneously by other species that represent similar resources that are physiologically equivalent. Indeed, Guimarães et al. [51] suggested that *Mg*. *migonei* is a permissive vector for *L*. *infantum*, and Oliveira et al. [52] reported an experimental infection rate of 10.6% for *L*. *infantum* in *Lu*. *cruzi*. It is apparent that sand fly species in general present the necessary conditions to act as host, and likely as vector, for *L*. *infantum*. It must be remembered that evidence of infection by molecular methods does not imply that the sand fly is capable of transmission. An important consequence of this new vision on the dynamics of pathogen life cycles is that epidemiological studies and programs should consider other potential host species in their methodologies. For *L*. *infantum* and other species of the genus, other sand fly species besides *Lu*. *longipalpis* should be considered as potential disease vectors. Thus, it is quite conceivable that VL transmission can occur in *Lu*. *longipalpis* absence. Our results present circumstantial evidences that these species can act as *L*. *infantum* vectors in the triple-border areas. Complementary studies are clearly required to test the hypothesis of *L infantum* host switching. The capacity of this parasite species to use other sand fly species as vector has a great significance in respect to VL control programs in tropical America. Ignoring the possibility of alternative *L*. *infantum* life cycles involving other vectors and sources of infection (including other mammal species) in new transmission areas is a dangerous assumption if VL control is desired.

Besides VL, our results also present concerns about the CL. Of 179 female sand fly examined by PCR-RFLP, 64 produced *Leishmania* patterns and the sequencing confirmed their identifications as *L*. *infantum* and *L*. *braziliensis*. This is the first record of *L*. *braziliensis* in this region of Brazil in these vectors. Therefore, two species appear to be circulating sympatrically and probably syntopically. In this work, we observed *Ny*. *whitmani*, previously reported only in rural areas, infected by two *Leishmania* species (i.e., *L*. *infantum* and *L*. *braziliensis*) in rural, ruro-urban and urban areas. It is necessary that the municipal and state authorities be aware and attentive to the possible adaptation of these vectors in urban and peri-urban areas. The vector studies carried out on the Brazilian side of the triple border revealed the existence of a transmission scenario for both forms of leishmaniases (cutaneous and visceral). This must be considered seriously by the health services. First, adequate diagnosis and treatment must be available to avoid mutilating sequels in undiagnosed CL individuals or patients who are treated incorrectly, articulated urban planning with the neighbouring cities is essential for the efficient control of leishmaniases.

### Public health in the triple border

Regarding environmental issues, it should be noted that all human activity generates environmental impacts, which compromise the equilibrium and the existing state of an environment. These impacts are man-made according to their needs, which vary in intensity and speed over time [53]. Considering the health concerns, these issues condition and/or intensify environmental vulnerabilities, thus amplifying the possibility of establishing certain types of diseases. The risk generated by man comes from the change in opportunity associated with the modernization process, generating technological and organizational innovations. These factors promote the production of wealth, inequalities and, consequently, unequal environmental risks in the society, if analyzed in micro scales. On the other hand, the risks generated by socioeconomic inequalities can produce a boomerang effect, since people from different social classes can suffer from the consequences of the modernization processes, such as infectious diseases related to elevated demographic concentration. In the last two decades, Brazil has been experiencing the expansion and urbanization of VL not only in large, such as Belo Horizonte and Campo Grande, but also in medium-size cities such as Araçatuba and Governador Valadares. The process of VL´s urbanization has been registered in four of Brazil´s five regions: Northeast (São Luís, Natal and Aracaju), North (Boa Vista and Santarém), Southeast (Belo Horizonte and Montes Claros) and Center West (Cuiabá and Campo Large) [54]. These scenarios have many common features that may be related to the dispersion of sand fly species.

Brazil's agrarian structure underwent deep modifications, associated with environmental and climate changes, reduction of investments in health and education, discontinuity of control actions, adaptation of the vectors to man-made environments, and difficulties in disease control in large urban agglomerates. In the triple frontier, it is important to emphasize that the issues are not only established in the socioeconomic sphere, because the study region presents a great ethnic-cultural discontinuity [55]. The social relationships of immigrants with the country of origin continue even if the individual has moved a long time ago, thus confronting the way of life of the city in which he has established himself [56]. The way of life of the population constitutes a decisive factor for the sanitary campaigns of waste control. Public authorities accept that a population´s way of life can be changed using information from long-term studies. However, this is complicated in cities where there is a high turnover of the inhabitants coming from neighbouring countries. These conditions can make it more difficult to execute successfully preventive health campaigns. This is where articulated urban planning between neighbouring cities, such as the three of the triple border can help to promote conjoint actions in the fight against vector borne diseases, such as the leishmaniases. There are regional and federal policies that are especially linked to MERCOSUL, that advocate intersectoral and intermunicipal articulation. However, there are problems in creating and maintaining programs for the prevention of vector-borne diseases that function in international border areas. It must be emphasized that for border municipalities, health planning needs to project beyond the municipal, state, and federal administrative limits.

The emergence of autochthonous VL in Paraná, more specifically in FI and STI, raises the concern of how international borders relate to the appearance of diseases. Farmer [55], stated that the ineffectiveness of political boundaries helps the spread of pathogenic microorganisms. For this author, political boundaries function as semipermeable membranes, open to the frequency of circulation of diseases, and closed to the free circulation of medicines. This fact demonstrates how countries' governments still do not consider their borders as a gateway to communicable diseases. The dynamics of many emerging diseases are not established within a single national territory and control is insufficient if realized by a single nation-state. However, the author does not ignore the effect of boundaries and frontiers in the spread of diseases and proposes further studies on intercountry borders [57]. The social inequalities of neighbouring countries must be considered beyond a critical sociology which can define the true transnational borders of pandemics.

Summary: We have shown that endemic visceral leishmaniases are present on the Brazilian side of triple border. There is therefore a potential risk that this disease could spread to other municipalities of Paraná state, as well as to the neighbouring states or countries, resulting in the installation of new foci. Many urban areas of tropical America are propitious for the establishment of *Lu*. *longipalpis* and without exception they have large dog populations. PAHO´s initiative in supporting the public health policies in the border areas of this study is laudable. This action is crucial but if stakeholders do not act quickly in controlling leishmaniases in this region as the scenario will inevitably become worse.
